# *N*-(5-Bromo-2-chloro­benz­yl)-*N*-cyclo­propyl­naphthalene-2-sulfonamide

**DOI:** 10.1107/S1600536809014457

**Published:** 2009-04-22

**Authors:** C. Suneel Manohar Babu, Helen P. Kavitha, R. Arulmozhi, Jasmine P. Vennila, V. Manivannan

**Affiliations:** a Nicholas Piramal Research Centre, Nicholas Piramal India Limited, Mumbai 400 063, India; b Department of Chemistry, SRM University, Ramapuram, Chennai 600 089, India; c Department of Chemistry, SRM University, Kattankulathur Campus, Kanchipuram, India; d Department of Physics, Panimalar Institute of Technology, Chennai 600 095, India; e Department of Research and Development, PRIST University, Vallam, Thanjavur 613 403, India

## Abstract

In the title compound, C_20_H_17_BrClNO_2_S, the dihedral angle between the benzene ring and the naphthalene plane is 8.95 (8)°. The crystal packing is stabilized by weak inter­molecular C—H⋯O, C—H⋯Cl and π–π [centroid–centroid distance = 3.8782 (16) Å] inter­actions.

## Related literature

For biological activity, see: Li *et al.* (1995 ▶); Maren (1976 ▶); Misra *et al.* (1982 ▶); Yoshino *et al.* (1992 ▶). For related structures, see: Ramachandran *et al.* (2008 ▶); Vennila *et al.* (2009 ▶). For graph-set notation, see: Bernstein *et al.* (1995 ▶).
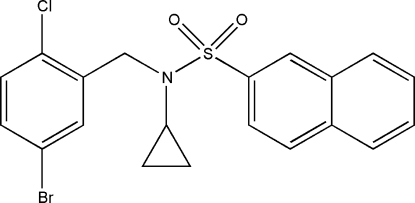


## Experimental

### 

#### Crystal data

C_20_H_17_BrClNO_2_S
*M* *_r_* = 450.77
Monoclinic, 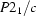

*a* = 12.1759 (5) Å
*b* = 7.5881 (3) Å
*c* = 20.5752 (8) Å
β = 95.393 (1)°
*V* = 1892.57 (13) Å^3^
*Z* = 4
Mo *K*α radiation
μ = 2.44 mm^−1^
*T* = 295 K
0.22 × 0.18 × 0.14 mm

#### Data collection

Bruker KappaAPEXII diffractometer
Absorption correction: multi-scan (*SADABS*; Sheldrick, 1996 ▶) *T*_min_ = 0.616, *T*_max_ = 0.727
23845 measured reflections
5240 independent reflections
3528 reflections with *I* > 2σ(*I*)
*R*_int_ = 0.029

#### Refinement

*R*[*F* ^2^ > 2σ(*F* ^2^)] = 0.039
*wR*(*F* ^2^) = 0.103
*S* = 1.01
5240 reflections
235 parameters
H-atom parameters constrained
Δρ_max_ = 0.71 e Å^−3^
Δρ_min_ = −0.81 e Å^−3^

### 

Data collection: *APEX2* (Bruker, 2004 ▶); cell refinement: *SAINT* (Bruker, 2004 ▶); data reduction: *SAINT*; program(s) used to solve structure: *SHELXS97* (Sheldrick, 2008 ▶); program(s) used to refine structure: *SHELXL97* (Sheldrick, 2008 ▶); molecular graphics: *PLATON* (Spek, 2009 ▶); software used to prepare material for publication: *SHELXL97*.

## Supplementary Material

Crystal structure: contains datablocks global, I. DOI: 10.1107/S1600536809014457/gk2206sup1.cif

Structure factors: contains datablocks I. DOI: 10.1107/S1600536809014457/gk2206Isup2.hkl

Additional supplementary materials:  crystallographic information; 3D view; checkCIF report

## Figures and Tables

**Table 1:** Hydrogen-bond geometry (Å, °)

*D*—H⋯*A*	*D*—H	H⋯*A*	*D*⋯*A*	*D*—H⋯*A*
C8—H8⋯Cl1^i^	0.98	2.79	3.612 (3)	142
C12—H12⋯O2^ii^	0.93	2.36	3.231 (3)	156

Symmetry codes: (i) 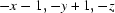; (ii) 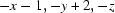.

**Fig. 1.**
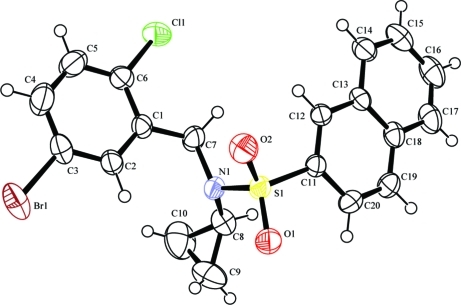
The molecular structure of the title compound, with atom labels and 50% probability displacement ellipsoids for non-H atoms.

**Fig. 2.**
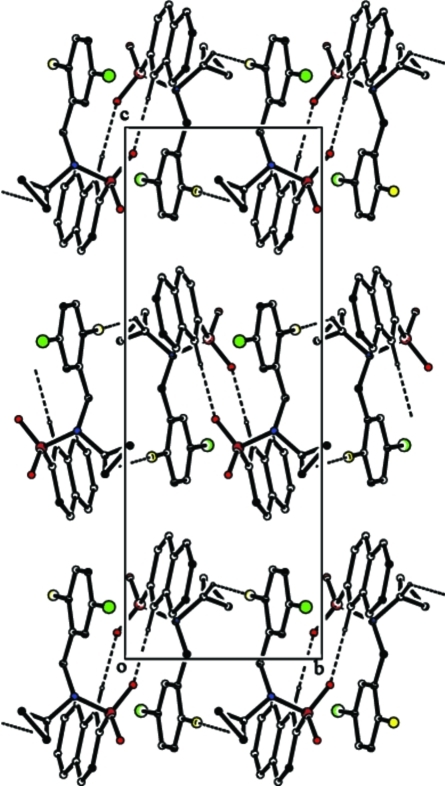
The packing of the title compound, viewed down the a axis. H-bonds are shown as dashed lines; H atoms not involved in hydrogen bonding have been omitted.

## supplementary crystallographic information

### Comment

Sulfonamides exhibit antibacterial (Misra *et al.*, 1982), insulin-releasing (Maren, 1976), anti-inflammatory (Li *et al.*, 1995) and antitumor (Yoshino *et al.*, 1992) activities. The geometric parameters in the title compound agree with the reported values of similar structure (Ramachandran *et al.*, 2008; Vennila *et al.*, 2009).

The dihedral angle between the phenyl ring and naphthalene ring is 8.95 (8)°. The geometry around S1 atom is distorted from a regular tetrahedron [O1—S1—N1 = 107.09 (10) °; O2—S1—N1 = 105.66 (11)°; O1—S1—C11 = 108.25 (10)°]. The molecular structure is stabilized by weak intramolecular C—H···O and C—H···N interactions and the crystal packing is stabilized by a weak intermolecular C—H···O, C—H···Cl (Fig. 2) and π–π [*Cg*2···*Cg*4(-1 - *x*, 1 - *y*, -*z*) = 3.8782 (16) Å; *Cg*2-centroid of ring C1–C6; *Cg*4-centroid of C13–C18 ring] interactions.

The intermolecular C8—H8···Cl1 interaction generates 14-membered ring, with graph-set motif *R*_2_^2^(14) and C16—H16···O2 interaction generates ten-membered ring, with graph-set motif *R*_2_^2^(10).

### Experimental

1 g (3.6 mmol) of 5-bromo-2-chloro-benzyl-cyclopropyl-amine is dissolved in 20 ml of ethyl acetate. To the above mixture, 0.57 g (7.2 mmol) of pyridine is added with stirring and then 0.7 g (3 mmol) of naphthalene-2-sulfonyl chloride is added and heated to 50 °C for 6 h. The reaction mass is cooled to room temperature and 20 ml of water is added. The aqueous layer is separated. The ethyl acetate layer is washed twice with 10% sodium chloride solution and dried over 2 g of anhydrous sodium sulfate. The solvent is removed under vacuum and the crude product obtained is recrystallized from hexane–ethyl acetate mixture to get diffraction quality white crystals.

### Refinement

H atoms were positioned geometrically and refined using riding model with C—H = 0.93–0.98 Å and *U*_iso_(H) = 1.2Ueq(C) for aryl and methine H atoms and *U*_iso_(H) = 1.5Ueq(C) for methylene H atoms.

### Crystal data

**Table:**

C_20_H_17_BrClNO_2_S	*F*(000) = 912
*M**_r_* = 450.77	*D*_x_ = 1.582 Mg m^−^^3^
Monoclinic, *P*2_1_/*c*	Mo *K*α radiation, λ = 0.71073 Å
Hall symbol: -P 2ybc	Cell parameters from 6275 reflections
*a* = 12.1759 (5) Å	θ = 2.5–29.7°
*b* = 7.5881 (3) Å	µ = 2.44 mm^−^^1^
*c* = 20.5752 (8) Å	*T* = 295 K
β = 95.393 (1)°	Block, colourless
*V* = 1892.57 (13) Å^3^	0.22 × 0.18 × 0.14 mm
*Z* = 4	

### Data collection

**Table:**

Bruker KappaAPEXII diffractometer	5240 independent reflections
Radiation source: fine-focus sealed tube	3528 reflections with *I* > 2σ(*I*)
graphite	*R*_int_ = 0.029
ω and φ scans	θ_max_ = 29.7°, θ_min_ = 2.5°
Absorption correction: multi-scan (*SADABS*; Sheldrick, 1996)	*h* = −16→16
*T*_min_ = 0.616, *T*_max_ = 0.727	*k* = −9→10
23845 measured reflections	*l* = −28→26

### Refinement

**Table:**

Refinement on *F*^2^	Primary atom site location: structure-invariant direct methods
Least-squares matrix: full	Secondary atom site location: difference Fourier map
*R*[*F*^2^ > 2σ(*F*^2^)] = 0.039	Hydrogen site location: inferred from neighbouring sites
*wR*(*F*^2^) = 0.103	H-atom parameters constrained
*S* = 1.01	*w* = 1/[σ^2^(*F*_o_^2^) + (0.0433*P*)^2^ + 1.1018*P*] where *P* = (*F*_o_^2^ + 2*F*_c_^2^)/3
5240 reflections	(Δ/σ)_max_ < 0.001
235 parameters	Δρ_max_ = 0.71 e Å^−^^3^
0 restraints	Δρ_min_ = −0.81 e Å^−^^3^

### Fractional atomic coordinates and isotropic or equivalent isotropic displacement parameters (Å^2^)

**Table:**

	*x*	*y*	*z*	*U*_iso_*/*U*_eq_	
Br1	0.04003 (2)	0.91779 (4)	0.097687 (17)	0.06646 (13)	
Cl1	−0.44538 (6)	0.63579 (10)	0.11975 (3)	0.05481 (18)	
S1	−0.36612 (5)	0.92504 (8)	−0.10261 (2)	0.03567 (13)	
N1	−0.31258 (15)	0.7424 (3)	−0.07080 (8)	0.0388 (4)	
O1	−0.30118 (14)	0.9756 (3)	−0.15345 (8)	0.0490 (4)	
O2	−0.37562 (15)	1.0407 (3)	−0.04900 (8)	0.0528 (5)	
C1	−0.27720 (18)	0.7338 (3)	0.05050 (10)	0.0360 (5)	
C2	−0.17142 (18)	0.7960 (3)	0.04688 (11)	0.0411 (5)	
H2	−0.1449	0.8125	0.0064	0.049*	
C3	−0.1051 (2)	0.8337 (4)	0.10303 (13)	0.0465 (6)	
C4	−0.1415 (2)	0.8122 (4)	0.16319 (13)	0.0607 (8)	
H4	−0.0957	0.8388	0.2006	0.073*	
C5	−0.2464 (2)	0.7508 (4)	0.16785 (12)	0.0583 (7)	
H5	−0.2722	0.7350	0.2086	0.070*	
C6	−0.31283 (19)	0.7129 (3)	0.11212 (11)	0.0405 (5)	
C7	−0.3536 (2)	0.6863 (4)	−0.00886 (11)	0.0484 (6)	
H7A	−0.3642	0.5596	−0.0098	0.058*	
H7B	−0.4249	0.7406	−0.0052	0.058*	
C8	−0.2972 (2)	0.6020 (3)	−0.11672 (13)	0.0488 (6)	
H8	−0.3642	0.5537	−0.1402	0.059*	
C9	−0.1969 (3)	0.6016 (5)	−0.15207 (18)	0.0736 (10)	
H9A	−0.2033	0.5561	−0.1963	0.088*	
H9B	−0.1454	0.6984	−0.1441	0.088*	
C10	−0.2075 (3)	0.4754 (5)	−0.0985 (2)	0.0897 (12)	
H10A	−0.1625	0.4949	−0.0577	0.108*	
H10B	−0.2203	0.3526	−0.1099	0.108*	
C11	−0.49910 (18)	0.8701 (3)	−0.13796 (10)	0.0345 (5)	
C20	−0.5150 (2)	0.8332 (4)	−0.20527 (10)	0.0441 (6)	
H20	−0.4573	0.8467	−0.2314	0.053*	
C19	−0.6153 (2)	0.7776 (4)	−0.23142 (11)	0.0472 (6)	
H19	−0.6263	0.7558	−0.2760	0.057*	
C18	−0.70337 (19)	0.7521 (3)	−0.19266 (11)	0.0421 (5)	
C13	−0.68651 (18)	0.7903 (3)	−0.12488 (11)	0.0365 (5)	
C12	−0.58277 (18)	0.8532 (3)	−0.09876 (10)	0.0352 (5)	
H12	−0.5714	0.8832	−0.0548	0.042*	
C17	−0.8073 (2)	0.6872 (4)	−0.21798 (14)	0.0573 (7)	
H17	−0.8205	0.6641	−0.2624	0.069*	
C16	−0.8879 (2)	0.6582 (4)	−0.17825 (16)	0.0655 (8)	
H16	−0.9558	0.6147	−0.1957	0.079*	
C15	−0.8704 (2)	0.6928 (4)	−0.11131 (15)	0.0590 (7)	
H15	−0.9263	0.6700	−0.0846	0.071*	
C14	−0.7729 (2)	0.7593 (4)	−0.08508 (13)	0.0476 (6)	
H14	−0.7628	0.7846	−0.0407	0.057*	

### Atomic displacement parameters (Å^2^)

**Table:**

	*U*^11^	*U*^22^	*U*^33^	*U*^12^	*U*^13^	*U*^23^
Br1	0.03377 (15)	0.0702 (2)	0.0924 (2)	−0.00308 (13)	−0.00958 (14)	0.00913 (17)
Cl1	0.0471 (4)	0.0642 (4)	0.0555 (3)	−0.0071 (3)	0.0171 (3)	0.0071 (3)
S1	0.0333 (3)	0.0404 (3)	0.0328 (2)	−0.0043 (2)	0.0008 (2)	0.0009 (2)
N1	0.0352 (10)	0.0467 (12)	0.0339 (8)	−0.0017 (9)	0.0002 (7)	0.0075 (8)
O1	0.0417 (9)	0.0582 (12)	0.0474 (9)	−0.0087 (8)	0.0056 (7)	0.0160 (8)
O2	0.0506 (11)	0.0555 (12)	0.0511 (9)	−0.0067 (9)	−0.0021 (8)	−0.0184 (8)
C1	0.0338 (11)	0.0356 (13)	0.0379 (10)	0.0029 (9)	0.0001 (8)	0.0086 (9)
C2	0.0325 (11)	0.0468 (15)	0.0434 (11)	0.0000 (10)	0.0009 (9)	0.0110 (10)
C3	0.0341 (12)	0.0456 (15)	0.0578 (14)	0.0034 (11)	−0.0066 (10)	0.0072 (12)
C4	0.0596 (17)	0.074 (2)	0.0452 (13)	−0.0058 (15)	−0.0117 (12)	0.0014 (14)
C5	0.0649 (18)	0.073 (2)	0.0376 (12)	−0.0087 (16)	0.0059 (12)	0.0004 (13)
C6	0.0394 (12)	0.0396 (14)	0.0432 (11)	0.0007 (10)	0.0065 (9)	0.0068 (10)
C7	0.0396 (13)	0.0652 (18)	0.0394 (11)	−0.0108 (12)	−0.0004 (10)	0.0158 (12)
C8	0.0416 (14)	0.0456 (16)	0.0587 (14)	0.0003 (11)	0.0016 (11)	0.0001 (12)
C9	0.0554 (19)	0.076 (2)	0.093 (2)	0.0054 (16)	0.0246 (17)	−0.0170 (19)
C10	0.090 (3)	0.073 (3)	0.103 (3)	0.033 (2)	−0.004 (2)	−0.003 (2)
C11	0.0329 (11)	0.0381 (13)	0.0317 (9)	0.0020 (9)	−0.0007 (8)	−0.0017 (9)
C20	0.0456 (13)	0.0547 (16)	0.0322 (10)	−0.0004 (12)	0.0045 (9)	−0.0022 (10)
C19	0.0485 (14)	0.0599 (17)	0.0317 (10)	0.0022 (12)	−0.0042 (10)	−0.0084 (11)
C18	0.0380 (12)	0.0424 (14)	0.0439 (12)	0.0037 (10)	−0.0070 (10)	−0.0073 (10)
C13	0.0316 (11)	0.0343 (13)	0.0432 (11)	0.0044 (9)	0.0018 (9)	−0.0033 (10)
C12	0.0339 (11)	0.0393 (13)	0.0320 (9)	0.0051 (10)	0.0008 (8)	−0.0054 (9)
C17	0.0422 (14)	0.0650 (19)	0.0612 (15)	0.0001 (13)	−0.0141 (12)	−0.0118 (14)
C16	0.0355 (14)	0.070 (2)	0.087 (2)	−0.0029 (14)	−0.0123 (14)	−0.0122 (17)
C15	0.0324 (13)	0.063 (2)	0.082 (2)	0.0015 (13)	0.0083 (13)	0.0011 (15)
C14	0.0373 (13)	0.0525 (16)	0.0534 (13)	0.0046 (12)	0.0059 (10)	−0.0023 (12)

### Geometric parameters (Å, °)

**Table:**

Br1—C3	1.891 (3)	C9—C10	1.475 (5)
Cl1—C6	1.738 (2)	C9—H9A	0.9700
S1—O1	1.4220 (16)	C9—H9B	0.9700
S1—O2	1.4229 (17)	C10—H10A	0.9700
S1—N1	1.641 (2)	C10—H10B	0.9700
S1—C11	1.761 (2)	C11—C12	1.363 (3)
N1—C8	1.448 (3)	C11—C20	1.409 (3)
N1—C7	1.475 (3)	C20—C19	1.356 (3)
C1—C2	1.380 (3)	C20—H20	0.9300
C1—C6	1.387 (3)	C19—C18	1.408 (3)
C1—C7	1.508 (3)	C19—H19	0.9300
C2—C3	1.376 (3)	C18—C17	1.411 (3)
C2—H2	0.9300	C18—C13	1.420 (3)
C3—C4	1.363 (4)	C13—C12	1.409 (3)
C4—C5	1.371 (4)	C13—C14	1.412 (3)
C4—H4	0.9300	C12—H12	0.9300
C5—C6	1.370 (4)	C17—C16	1.353 (4)
C5—H5	0.9300	C17—H17	0.9300
C7—H7A	0.9700	C16—C15	1.399 (4)
C7—H7B	0.9700	C16—H16	0.9300
C8—C10	1.475 (4)	C15—C14	1.355 (4)
C8—C9	1.478 (4)	C15—H15	0.9300
C8—H8	0.9800	C14—H14	0.9300
			
O1—S1—O2	119.67 (12)	C8—C9—H9A	117.8
O1—S1—N1	107.09 (10)	C10—C9—H9B	117.8
O2—S1—N1	105.66 (11)	C8—C9—H9B	117.8
O1—S1—C11	108.25 (10)	H9A—C9—H9B	114.9
O2—S1—C11	109.11 (11)	C9—C10—C8	60.1 (2)
N1—S1—C11	106.27 (11)	C9—C10—H10A	117.8
C8—N1—C7	115.3 (2)	C8—C10—H10A	117.8
C8—N1—S1	115.61 (15)	C9—C10—H10B	117.8
C7—N1—S1	115.72 (17)	C8—C10—H10B	117.8
C2—C1—C6	117.5 (2)	H10A—C10—H10B	114.9
C2—C1—C7	123.1 (2)	C12—C11—C20	121.5 (2)
C6—C1—C7	119.4 (2)	C12—C11—S1	119.14 (15)
C3—C2—C1	120.2 (2)	C20—C11—S1	119.26 (17)
C3—C2—H2	119.9	C19—C20—C11	119.2 (2)
C1—C2—H2	119.9	C19—C20—H20	120.4
C4—C3—C2	121.5 (2)	C11—C20—H20	120.4
C4—C3—Br1	118.6 (2)	C20—C19—C18	121.6 (2)
C2—C3—Br1	119.94 (19)	C20—C19—H19	119.2
C3—C4—C5	119.3 (2)	C18—C19—H19	119.2
C3—C4—H4	120.4	C19—C18—C17	122.9 (2)
C5—C4—H4	120.4	C19—C18—C13	118.7 (2)
C6—C5—C4	119.6 (2)	C17—C18—C13	118.4 (2)
C6—C5—H5	120.2	C12—C13—C14	121.7 (2)
C4—C5—H5	120.2	C12—C13—C18	119.0 (2)
C5—C6—C1	122.0 (2)	C14—C13—C18	119.3 (2)
C5—C6—Cl1	118.39 (19)	C11—C12—C13	119.95 (19)
C1—C6—Cl1	119.60 (18)	C11—C12—H12	120.0
N1—C7—C1	113.43 (19)	C13—C12—H12	120.0
N1—C7—H7A	108.9	C16—C17—C18	120.7 (3)
C1—C7—H7A	108.9	C16—C17—H17	119.7
N1—C7—H7B	108.9	C18—C17—H17	119.7
C1—C7—H7B	108.9	C17—C16—C15	120.9 (3)
H7A—C7—H7B	107.7	C17—C16—H16	119.6
N1—C8—C10	116.8 (3)	C15—C16—H16	119.6
N1—C8—C9	119.1 (2)	C14—C15—C16	120.6 (3)
C10—C8—C9	59.9 (2)	C14—C15—H15	119.7
N1—C8—H8	116.4	C16—C15—H15	119.7
C10—C8—H8	116.4	C15—C14—C13	120.2 (2)
C9—C8—H8	116.4	C15—C14—H14	119.9
C10—C9—C8	59.9 (2)	C13—C14—H14	119.9
C10—C9—H9A	117.8		
			
O1—S1—N1—C8	−52.60 (19)	N1—C8—C10—C9	−109.7 (3)
O2—S1—N1—C8	178.78 (17)	O1—S1—C11—C12	−166.32 (19)
C11—S1—N1—C8	62.93 (19)	O2—S1—C11—C12	−34.6 (2)
O1—S1—N1—C7	168.24 (16)	N1—S1—C11—C12	78.9 (2)
O2—S1—N1—C7	39.62 (19)	O1—S1—C11—C20	17.9 (2)
C11—S1—N1—C7	−76.22 (18)	O2—S1—C11—C20	149.7 (2)
C6—C1—C2—C3	0.4 (4)	N1—S1—C11—C20	−96.8 (2)
C7—C1—C2—C3	−178.7 (2)	C12—C11—C20—C19	−0.8 (4)
C1—C2—C3—C4	−0.4 (4)	S1—C11—C20—C19	174.9 (2)
C1—C2—C3—Br1	179.70 (19)	C11—C20—C19—C18	−1.5 (4)
C2—C3—C4—C5	0.3 (5)	C20—C19—C18—C17	−177.2 (3)
Br1—C3—C4—C5	−179.8 (2)	C20—C19—C18—C13	1.6 (4)
C3—C4—C5—C6	−0.3 (5)	C19—C18—C13—C12	0.4 (4)
C4—C5—C6—C1	0.3 (5)	C17—C18—C13—C12	179.3 (2)
C4—C5—C6—Cl1	−179.9 (2)	C19—C18—C13—C14	−177.7 (2)
C2—C1—C6—C5	−0.4 (4)	C17—C18—C13—C14	1.1 (4)
C7—C1—C6—C5	178.8 (3)	C20—C11—C12—C13	2.8 (4)
C2—C1—C6—Cl1	179.79 (19)	S1—C11—C12—C13	−172.87 (18)
C7—C1—C6—Cl1	−1.0 (3)	C14—C13—C12—C11	175.5 (2)
C8—N1—C7—C1	120.9 (2)	C18—C13—C12—C11	−2.6 (3)
S1—N1—C7—C1	−99.8 (2)	C19—C18—C17—C16	177.3 (3)
C2—C1—C7—N1	−10.4 (4)	C13—C18—C17—C16	−1.5 (4)
C6—C1—C7—N1	170.4 (2)	C18—C17—C16—C15	0.4 (5)
C7—N1—C8—C10	−68.4 (3)	C17—C16—C15—C14	1.2 (5)
S1—N1—C8—C10	152.3 (2)	C16—C15—C14—C13	−1.6 (4)
C7—N1—C8—C9	−137.2 (3)	C12—C13—C14—C15	−177.7 (3)
S1—N1—C8—C9	83.5 (3)	C18—C13—C14—C15	0.4 (4)
N1—C8—C9—C10	105.9 (3)		

### Hydrogen-bond geometry (Å, °)

**Table:**

*D*—H···*A*	*D*—H	H···*A*	*D*···*A*	*D*—H···*A*
C2—H2···N1	0.93	2.52	2.864 (3)	102
C20—H20···O1	0.93	2.56	2.926 (3)	104
C8—H8···Cl1^i^	0.98	2.79	3.612 (3)	142
C12—H12···O2^ii^	0.93	2.36	3.231 (3)	156

Symmetry codes: (i) −*x*−1, −*y*+1, −*z*; (ii) −*x*−1, −*y*+2, −*z*.

## References

[bb1] Bernstein, J., Davis, R. E., Shimoni, L. & Chang, N.-L. (1995). *Angew. Chem. Int. Ed. Engl.* **34**, 1555–1573.

[bb2] Bruker (2004). *APEX2* and *SAINT* Bruker AXS Inc., Madison, Wisconsin, USA.

[bb3] Li, J. J., Anderson, D., Burton, E. G., Cogburn, J. N., Collins, J. T., Garland, D. J., Gregory, S. A., Huang, H. C., Isakson, P. C., Koboldt, C. M., Logusch, E. W., Norton, M. B., Perkins, W. E., Reinhard, E. J., Seibert, K., Veenhuizem, A. W., Zang, Y. & Reitz, D. B. (1995). *J. Med. Chem.* **38**, 4570–4570. 10.1021/jm00022a023 7473585

[bb4] Maren, T. H. (1976). *Annu. Rev. Pharmacol. Toxicol.* **16**, 309–309. 10.1146/annurev.pa.16.040176.001521 59572

[bb5] Misra, V. S., Saxena, V. K. & Srivastava, R. J. (1982). *J. Indian Chem. Soc.* **59**, 781–781.

[bb6] Ramachandran, G., Kanakam, C. C. & Manivannan, V. (2008). *Acta Cryst.* E**64**, o873. 10.1107/S1600536808010052 PMC2961114 21202359

[bb7] Sheldrick, G. M. (1996). *SADABS* University of Göttingen, Germany.

[bb8] Sheldrick, G. M. (2008). *Acta Cryst.* A**64**, 112–122. 10.1107/S0108767307043930 18156677

[bb9] Spek, A. L. (2009). *Acta Cryst.* D**65**, 148–155. 10.1107/S090744490804362X PMC2631630 19171970

[bb10] Vennila, J. P., Kavitha, H. P., Thiruvadigal, D. J., Venkatraman, B. R. & Manivannan, V. (2009). *Acta Cryst.* E**65**, o72. 10.1107/S1600536808041032 PMC2967982 21581711

[bb11] Yoshino, H., Ueda, N., Niijma, J., Sugumi, H., Kotake, Y., Koyanagi, N., Yoshimatsu, K., Asada, M., Watanabe, T., Nagasu, T., Tsukahara, K., Lijima, A. & Kitoh, K. (1992). *J. Med. Chem.* **35**, 2496–2496. 10.1021/jm00091a018 1619621

